# Age and sex effects on DNA methylation sites linked to genes implicated in severe COVID-19 and SARS-CoV-2 host cell entry

**DOI:** 10.1371/journal.pone.0269105

**Published:** 2022-06-09

**Authors:** Jon Bohlin, Christian M. Page, Yunsung Lee, John H.-O. Pettersson, Astanand Jugessur, Per Magnus, Siri E. Håberg

**Affiliations:** 1 Department of Method Development and analytics, Norwegian Institute of Public Health, Oslo, Norway; 2 Center for Fertility and Health, Norwegian Institute of Public Health, Oslo, Norway; 3 Department of Mathematics, University of Oslo, Oslo, Norway; 4 Marie Bashir Institute for Infectious Diseases and Biosecurity, School of Life and Environmental Sciences and School of Medical Sciences, the University of Sydney, Sydney, New South Wales, Australia; 5 Zoonosis Science Center, Department of Medical Biochemistry and Microbiology, University of Uppsala, Uppsala, Sweden; 6 Department of Global Public Health and Primary Care, University of Bergen, Bergen, Norway; Chinese Academy of Sciences, CHINA

## Abstract

Male sex and advanced age are associated with severe symptoms of COVID-19. Sex and age also exhibit substantial associations with genome-wide DNA methylation (DNAm) differences in humans. Using a random sample of Illumina EPIC-based genome-wide methylomes from peripheral whole blood of 1,976 parents, participating in The Norwegian Mother, Father and Child Cohort Study (MoBa), we explored whether DNAm in genes linked to SARS-CoV-2 host cell entry and to severe COVID-19 were associated with sex and age. This was carried out by testing 1,572 DNAm sites (CpGs) located near 45 genes for associations with age and sex. We found that DNAm in 281 and 231 of 1,572 CpGs were associated (p_FDR_<0.01) with sex and aging, respectively. CpGs linked to SARS-CoV-2 host cell entry genes were all associated with age and sex, except for the *ACE2* receptor gene (located on the X-chromosome), which was only associated with sex (p_FDR_<0.01). Furthermore, we examined whether 1,487 autosomal CpGs associated with host-cell entry and severe COVID-19 were more or less associated with sex and age than what would be expected from the same number of randomly sampled genome-wide CpGs. We found that the CpGs associated with host-cell entry and severe COVID-19 were not more or less associated with sex (R^2^ = 0.77, p = 0.09) than the CpGs sampled from random genomic regions; age was actually found to be significantly less so (R^2^ = 0.36, p = 0.04). Hence, while we found wide-spread associations between sex and age at CpGs linked to genes implicated with SARS-CoV-2 host cell entry and severe COVID-19, the effect from the sum of these CpGs was not stronger than that from randomly sampled CpGs; for age it was significantly less so. These findings could suggest that advanced age and male sex may not be unsurmountable barriers for the SARS-CoV-2 virus to evolve increased infectiousness.

## Introduction

The clinical picture of coronavirus disease 2019 (COVID-19), caused by the severe acute respiratory syndrome coronavirus 2 (SARS-CoV-2) virus, shows substantial variation; from asymptomatic to severe pneumonia-like distress requiring the aid of respirators and, in some cases, even death [1]. The SARS-CoV-1 and SARS-CoV-2 Betacoronaviruses share the same host species, *i*.*e*. horseshoe bats (*Rhinolophus* sp.), in addition to several genetic and phenotypic similarities [2]. In humans, both viruses bind to a receptor, coded by a gene located on the X chromosome expressing an angiotensin converting enzyme (*ACE2*), with a trimeric Spike protein (S) [2]. The S protein is divided into the subdomains S1, which includes the receptor binding domain (RBD), and S2 that mediates viral-host membrane fusion [2] (See S1 Fig). Current knowledge suggests that SARS-CoV-2 can enter host cells via two routes [3, 4]. One route, the early entry pathway, is through virus-host cell membrane fusion. The other described route, known as the late entry pathway, is via virus attachment to the ACE2 receptor at the cell surface and endocytosis. Both modes of entry have in common that they are currently believed to exclusively go through the ACE2 receptor [3]. For the early entry pathway, the furin site, located between the S1 and S2 subdomains of the S protein, must be cleaved before the transmembrane serine 2 protease (TMPRSS2) can cleave the S2’ site. The cleavage of the furin site takes place during virus assembly and maturation [4]. The S2’ site is located within the S2 subdomain of the S protein and becomes exposed during binding to ACE2 if the furin site is cleaved [3]. Neuropilin-1 (NRP1) has recently been found to enhance SARS-CoV-2 infectivity through the early pathway entry but is not necessary for infection [5]. Cleavage of the furin site seems to complicate the late pathway entry as the S protein is not in an optimal state for entry via endocytosis [3]. Conversely, while complicating the early pathway entry due to the required cleavage, the uncleaved furin site increases affinity for the late pathway entry through endocytosis [3]. Cleavage of the furin site leads to a less stable S protein which seems to favor early versus late pathway entry. During the endocytosis phase of the late pathway entry, cathepsin L/B (CTSL and CTSB) cleave the S’ site before the viral genome is released into the cell for subsequent viral biosynthesis [3, 4].

Host infectivity to SARS-CoV-2 depends both on viral- and host genetic factors [6]. The gene coding for the ACE2 receptor is located on the X chromosome [7]. In humans, females have two copies of the X chromosome, inherited from each parent, while males have one maternally inherited copy [8]. The second copy of the X chromosome in females is replaced by a paternally inherited Y chromosome in males [9]. One of the two X chromosomes located in female cells is inactivated (XCI) which is marked by ubiquitous DNA methylation (DNAm) [8]. DNAm is typically taken to be the addition of a methyl group to Cytosine in Cytosine-Guanine (CpG) di-nucleotide pairs [10]. Which of the two X-chromosomes in females become inactivated varies randomly from cell to cell [8]. Hence, female cells express one of two ACE2 receptor variants inherited from each parents while males express only a maternally inherited variant [11, 12]. Due to the strong association with cell type differentiation [13], DNAm is the most studied epigenetic mark [10]. It has also been found that genome-wide DNAm varies throughout the life-course and as a consequence of genetics and environmental exposures [14]. In particular, a substantial fraction of genome-wide DNAm differences have both been attributed to age and sex differences in humans [15, 16]. Indeed, numerous DNAm-based epigenetic clocks have been created that can predict chronological age from different cell types [17]. Epigenetic clocks have also been discovered from certain cell types that can predict age with a high accuracy [18]. There are also epigenetic clocks that can measure biological age linked to all-cause mortality [19]. DNAm-based epigenetic clocks have recently been linked to T and NK cell activation suggesting that epigenetic clocks are in general reflective of host immune system status [20].

Current risk factors for severe COVID-19 symptoms are advanced age [21, 22] and being male [11]. COVID-19 outcomes may also be influenced by DNAm in multiple tissues and organs owing to the systemic nature of the disease [23]. Two recent genome-wide association studies (GWASs) of severe COVID-19 [1, 24] reported single nucleotide polymorphisms (SNPs) significantly linked to 16 genes in total. The strong association of severe COVID-19 with sex [11] and age [25] motivated us to examine whether DNAm differences associated with sex and age could be linked to genes implicated with severe COVID-19, defined as critical respiratory failure [1, 24]. We additionally scrutinized age and sex effects of CpGs related to genes involved in SARS-CoV-2 host cell entry. In particular, we examined both *ACE2* and *TMPRSS2* genes and their associated STRING network clusters [26] (11 + 11 = 22 genes in total) as well as 7 genes [3, 5, 27, 28] implicated with both early and late pathway entry.

In total, we looked for sex and age related DNAm differences in 1,572 CpG loci (1,487 autosomal CpGs and 85 X chromome CpGs), linked to 45 genes (41 autosomal genes and 4 X chromosome genes) associated with severe COVID-19 and SARS-CoV-2 host cell entry, in 1,976 parents participating in the Norwegian Mother, Father and Child Cohort Study (MoBa).

## Materials and methods

The study population was taken from The Norwegian Mother, Father and Child Study cohort (MoBa) [29]. Information regarding the study population can be found in Table 1.

**Table 1:** Study population characteristics.

	Males	Females
*Number*	991(50.2%)	985(49.8%)
*Median age in years (mean)*	32.3(32.8)(18.3, 58.6)	30.1(30.1)(18.3, 45.5)

The table shows the study population’s sex (number (percent)) and age (median (mean)(min,max)) characteristics.

The establishment of MoBa and initial data collection was based on a license from the Norwegian Data Protection Agency and approval from The Regional Committees for Medical and Health Research Ethics (REK). The MoBa cohort is currently regulated by the Norwegian Health Registry Act. The current study was approved by REK South-East (2017/1362) in Norway 26.09.2017. Data collection by MoBa was carried out during 2001–2009 in accordance with the Norwegian Data Protection Agency after securing approval from REK. All participants provided written informed consent.

We used 1,976 randomly sampled adults (see Table 1) from MoBa [29]. The DNA methylation of these subjects were measured from peripheral blood using Illumina’s Infinium MethylationEPIC array. Quality control and preprocessing was carried out using the RnBeads package [30]. During the quality control procedure, probes with cross-hybridization, high detection P-value (>0.01) and those near single-nucleotide polymorphisms (SNP) (three last bases of the probe sequence overlaps with a SNP) were excluded resulting in a total of 770,586 out of 846,232 autosomal probes (total probes on the Illumina EPIC array is 865,859 probes, 19,090 probes are located on the X chromosome and 537 on the Y chromosome). Signal intensities were background-corrected using enmix.oob [31] before probe Type I and Type II calibrations were carried out using the beta-mixture quantile normalization (BMIQ) procedure [32]. The X chromosomes were preprocessed independently of the autosomes to prevent bias due to X chromosome inactivation. The same guidelines were used for the X chromosomes as for the autosomes. Using the same exclusion principles as for autosomes we ended up with 16,841 probes on the X chromosome on which BMIQ was performed. Further details regarding quality control of these samples can be found in Lee et al. [33]. Estimates of the different cell types were carried out using the reference set described by Salas et al. [34] using the Houseman procedure [35]. All cell types, *i*.*e*. CD4+ T and CD8+ T cells, NK cells, B cells, monocytes and neutrophils, were adjusted for in the regression analyses.

Statistical modeling was carried out using mixed-effects regression with the R package ‘nlme’ [36, 37]. DNAm at each CpG was treated as the outcome (β values), with sex and age as explanatory variables. EPIC array plates were included as random intercept effects. Regression models used to assess the DNAm effects of sex and age were reciprocally adjusted for age and sex together with cell type proportion estimates.

Adjustment for multiple testing was performed using false discovery rate (FDR) and Bonferroni on 770,586 autosomal + 16,841 X chromosomal CpG sites = 787,427 tests in total. We considered only genome-wide p_FDR_<0.01 as statistically significant. Analyses of CpGs located on the X-chromosome (particularly CpGs linked to the *ACE2* receptor gene) were stratified for males and females due to XCI.

Prediction models were performed using Lasso penalized regression from the ‘glmnet’ package [38] with the X matrix consisting of the 1,487 CpGs linked to 41 genes associated with severe COVID-19 and SARS-CoV-2 host cell entry located on the autosomes. The outcome of the Lasso regression was binomial for sex (male/female) and continuous for age (days, subsequently converted to years). Half the individuals (n = 988) were selected randomly with the corresponding outcome (sex/age) for training while prediction was carried out on the other half (n = 988 individuals). Subsequent linear regressions were performed on predicted versus given age in days and sex respectively, from which the coefficient of variation R^2^ was recorded. For randomization, 1,487 CpGs were selected randomly from the autosomal CpGs with training and prediction performed on the same halves of the samples as above. The randomization procedure was repeated 1,000 times for both sex and age outcomes, respectively, with subsequent linear regressions on given and predicted age/sex. The variance explained parameter (R^2^) was recorded for the regression models and corresponding distributions were made as can be seen in Fig 2C and 2D for sex and age. The p values for R^2^ were computed using the empirical distributions shown in Fig 2 panels C and D. All estimations and regression results can be found in S1 Appendix.

## Results and discussion

In total, we examined 1,572 CpGs (including 85 CpGs on the X chromosome) related to 45 host genes (4 genes were located on the X chromosome). Because of X chromosome inactivation (XCI) [8], we analyzed genes located on autosomes separately from genes on the X chromosome. Results from the regression analyses can be found in Table 2 as well as in S1 Appendix, which also includes regression estimates and information regarding the genes included in the study.

**Table 2:** SARS-CoV-2 genes associated with host cell entry and severe COVID-19. The table shows information regarding genes associated with SARS-CoV-2 host cell entry as well as severe COVID-19. *Source* describes the source justifying the inclusion of the gene, *Gene identifier* refers to the gene symbol, *Sex associated* shows number of DMPs significantly associated with sex (p_FDR_<0.01), *Age associated* shows the same for age, *CpGs linked to gene* indicates the number of CpGs on the EPIC array linked to that particular gene and, finally, the last column *Chromosome* displays the chromosome number on which the gene is located.

Source	Gene	Sex associated	Age associated	CpGs linked to gene	Chromosome
ACE2 Cluster [12]	ACE2*	12	0	15	X
	AGT	0	1	13	1
	AGTR1	6	12	29	3
	AGTR2*	5	0	5	X
	DPP4	6	7	36	2
	MEP1A	5	1	12	6
	MEP1B	2	1	3	18
	MME	2	16	35	3
	PRCP	9	1	38	11
	REN	2	1	13	1
	XPNPEP2*	17	0	18	X
TMPRSS2 Cluster [12]	TMPRSS2	1	7	32	21
	AR*	47	0	47	X
	ERG	16	15	78	21
	ETV1	10	6	45	7
	ETV4	0	5	32	17
	FAM3B	1	7	18	21
	FKBP5	9	5	49	6
	NKX3-1	2	4	15	8
	PTEN	14	2	76	10
	SLC45A3	6	4	34	1
	TMPRSS4	3	10	44	11
GWAS [1]	ABO	0	2	18	9
Entry [14]	ADAM17	8	0	33	2
GWAS [11]	CCHCR1	5	2	59	6
GWAS [1]	CCR9	1	4	15	3
Entry (late pathway) [3]	CTSB	6	4	38	8
Entry (late pathway) [3]	CTSL	0	3	5	9
GWAS [1]	CXCR6	2	1	14	3
GWAS [11]	DPP9	5	1	52	19
Entry (early pathway) [3]	FURIN	7	5	47	15
GWAS [1]	FYCO1	5	4	38	3
GWAS [11]	HLA-G	6	8	18	6
Entry [13]	HMGB1	5	2	22	13
GWAS [1]	IFNAR2	2	1	29	21
GWAS [1,11]	LZTFL1	6	7	40	3
GWAS [11]	NOTCH4	18	38	139	6
Entry [4]	NRP1	13	14	79	10
Entry [4]	NRP2	4	19	85	2
GWAS [11]	OAS1	5	2	15	12
GWAS [11]	OAS2	1	1	23	12
GWAS [11]	OAS3	3	0	21	12
GWAS [1]	SLC6A20	1	7	39	3
GWAS [11]	TYK2	2	1	46	19
GWAS [1]	XCR1	1	0	10	3
**Total**	**45**	**281 (**200 without X chromosome genes)	**231**	**1572** (1487 on autosomes + 85 on X chromosome**)**	

‘*’ Gene located on X chromosome.

**Fig 1:**
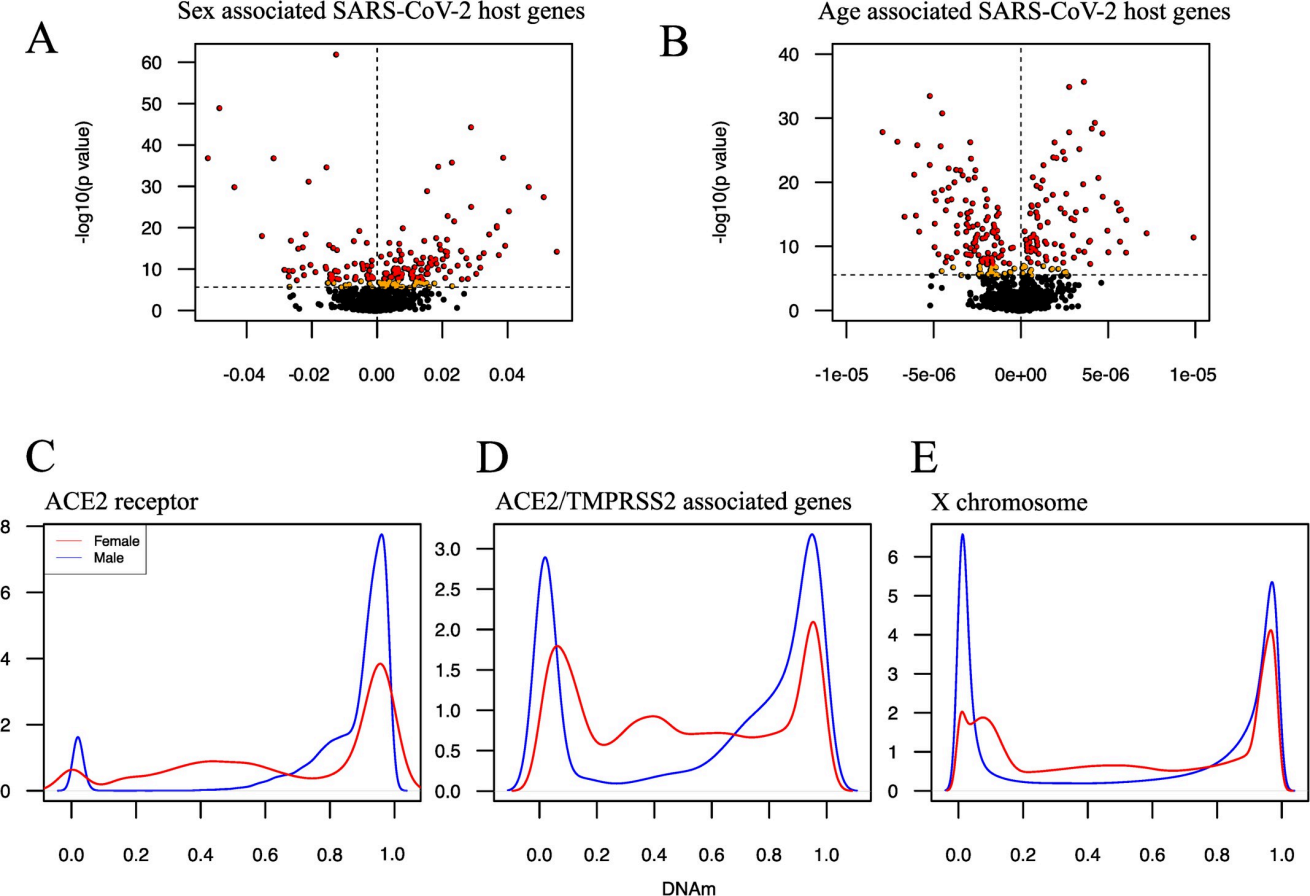
DNA methylation in genes associated with COVID-19. Panel A) shows a volcano plot of the–log_10_ transformed p values (vertical axis) of the coefficients resulting from regressing DNAm in autosomal CpGs linked to COVID-19 genes on sex (horizontal axis). Black colored dots signify non-significantly associated CpGs, orange dots p_FDR_<0.01 significantly associated CpGs and red dots Bonferroni p_B_<0.05 significantly associated CpGs. Panel B is similar to Panel A but the coefficients now reflect age instead of sex. The DNAm density of the CpGs linked to the *ACE2* receptor gene on the X chromosome can be seen in Panel C. Red colored lines designate females while blue lines males. Panel D shows the DNAm density for all CpGs related to the four *ACE2*/*TMPRSS2* STRING network clustered genes (*ACE2*, *AGTR2*, *AR*, *XPNPEP2*) located on the X chromosome, while the total DNAm density differences between males and females stemming from the 16,841 X-linked CpGs is depicted in Panel E.

We found that 200 and 231 differentially methylated probes (DMPs), located near genes related to both SARS-CoV-2 host cell entry and severe COVID-19, were significantly associated (p_FDR_<0.01) with sex and age respectively on autosomes (See Fig 1, panels A and B). While sex-associated CpGs (Fig 1A) were more hyper-methylated (800 vs 687 CpGs, 133 vs 69 DMPs p_FDR_<0.01) age-associated CpGs (Fig 1B) were predominantly hypo-methylated (839 vs 648 CpGs, 130 vs 101 DMPs p_FDR_<0.01). Linear regressions of DNAm of CpGs on the X-chromosome, assessing whether sex differences could be found at any of the sites connected to the genes linked with SARS-CoV-2 host cell entry (no genes currently identified with severe COVID-19 were located on the X chromosome), resulted in 81 significant DMPs (p_FDR_<0.01) out of 85. In general, there are profound DNAm differences between males and females on the X chromosome (Fig 1 panels C, D and E). Whether these are true differences or artifacts on the EPIC array resulting from XCI is unclear. We therefore performed stratified regression analysis for males and females with age as the explanatory variable and DNAm from each CpG as outcome (β values). Age was not significantly associated with any CpGs related to the *ACE2* gene or any other gene linked to SARS-CoV-2 host cell entry on the X chromosome. This may suggest that the SARS-CoV-2 virus may bind with equal affinity to the ACE2 host receptor in all age groups in peripheral blood. The CpGs on the EPIC array located near the *ACE2* gene are mainly found in transcription start sites (TSSs) (5 CpGs), untranslated regions (UTR’s)(2 CpGs), gene bodies (6 CpGs) and exons (2 CpGs) according to UCSC annotation (https://genome.ucsc.edu/). The five CpGs associated with the *ACE2* gene annotated to TSS were hyper-methylated, with some slight hemi-methylation in females that could be an artifact of XCI (see S2 Fig). This could indicate that the *ACE2* gene is not expressed in whole blood, which has been suggested elsewhere [39]. If, on the other hand, the *ACE2* gene is expressed, our findings may point to that the *ACE2* gene is differently methylated between males and females supporting the previously reported sex differences with regards to COVID-19 infection [11]. In the *ACE2* gene STRING network cluster we also find the *DPP4* receptor gene for the more deadly but far less infectious Middle Eastern Respiratory Syndrome Coronavirus (MERS-CoV) [2]. For the *DPP4* gene, 6 CpGs were significantly associated with sex and 7 with age. In contrast to the ACE2 receptor, the TMPRSS2 protease, linked to early pathway entry, is mostly associated with age (6 DMPs versus 1 DMP for sex). The furin protease had 7 DMPs associated with sex and 5 with age. For the genes coding for the proteases facilitating late entry pathway (*CTSL* and *CTSB*) we found that no CpGs were significantly associated with sex for *CTSL*, while 3 (of 5) CpGs were associated with age. For *CTSB*, 6 CpGs were significantly associated with sex and 4 with age. Other genes associated with SARS-CoV-2 host cell entry included metallopeptidase domain 17 (*ADAM17*) [28] (8 and 0 CpGs significantly associated with sex and age, respectively), high mobility group box 1 gene (*HMGB1*) [27] (5 and 2 DMPs), *NRP1* (13 and 14 DMPs) as well as *NRP2* genes [5] (4 and 19 DMPs). These findings may suggest that age could potentially influence the mode of entry for SARS-CoV-2 [40]. In particular, as the CpGs linked to the *TMPRSS2* gene were strongly associated with age our findings may suggest that enhanced late pathway entry via endocytosis could increase COVID-19 infectivity by allowing additional age groups to be more susceptible to infection [41].

Only one of 16 genes were shared between the two previously mentioned GWASs: Leucine zipper transcription factor-like proten 1 (LZTFL1). Of the DMPs linked to the *LZTFL1* gene, there were 6 CpGs significantly associated with sex and 7 with age. In total, we found that 63 CpGs were significantly associated with sex while 79 were associated with age of the 576 CpGs linked to the 16 genes associated with severe COVID-19.

**Fig 2:**
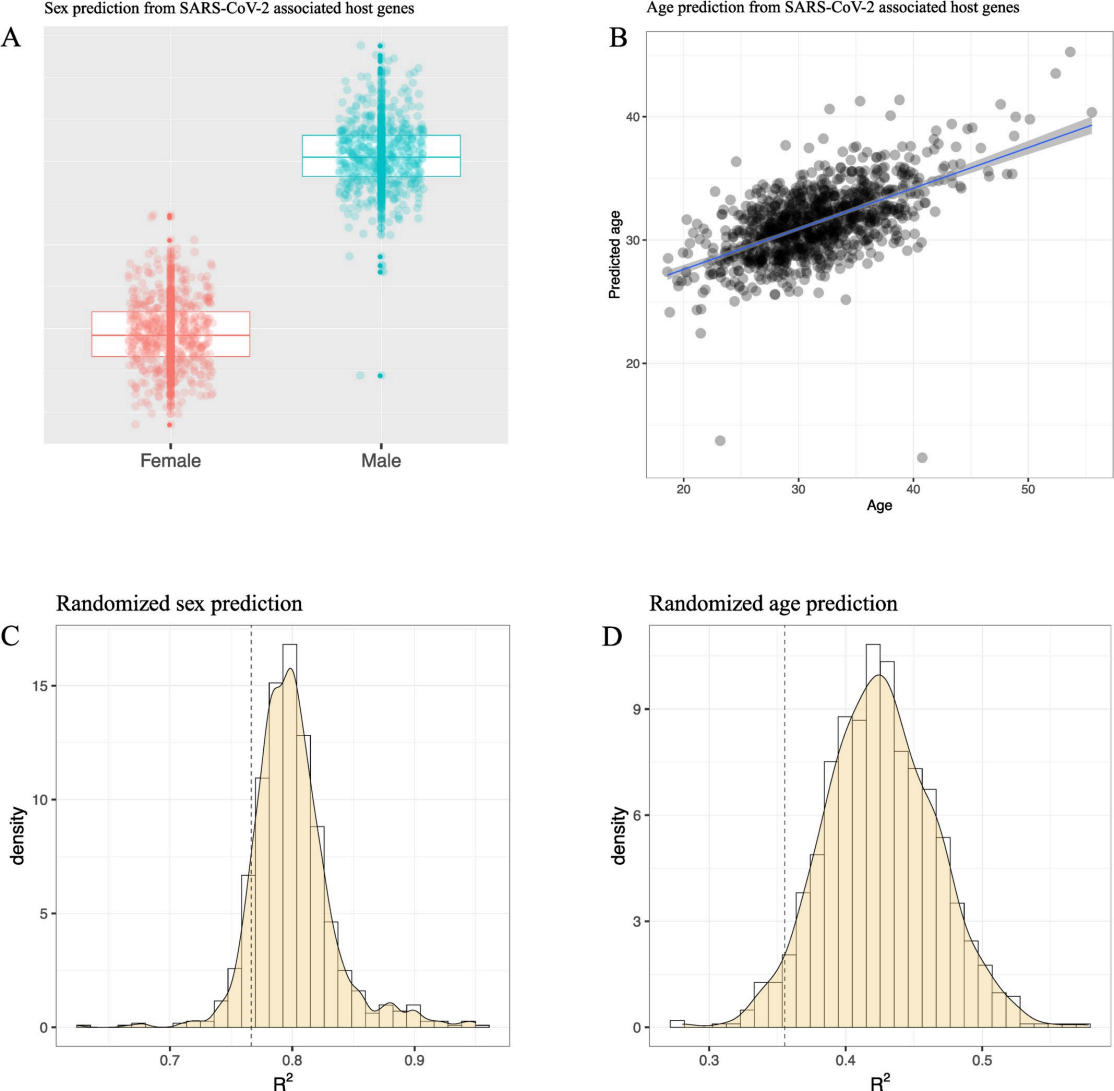
Predicting sex and age in SARS-CoV-2 associated host genes. Panel A shows the association between autosomal CpGs, linked to SARS-CoV-2 host genes/severe COVID-19, and predicted sex (vertical axis). Panel B shows the association between CpGs predicting age (vertical axis) and given age (horizontal axis). The distributions of the variance explained parameter R^2^, stemming from 1,000 randomizations of genome-wide randomly selected CpGs against sex and age, are shown in Panels C and D, respectively. The vertical dashed line designates the corresponding R^2^ for the sex (R^2^ = 0.77, p = 0.09) and age (R^2^ = 0.36, p = 0.04) regression models depicted in panels A and B.

Since it has been found that both sex and age DMPs are wide-spread in the human genome [15], we made sex- and age predictors based only on the 1,487 CpGs linked to the autosomal genes associated with SARS-CoV-2 host cell entry and severe COVID-19. We randomly selected half the samples for training of the Lasso classifier (n = 988 samples) and the other half (again n = 988 samples) for prediction. Fig 2, panels A and B, show the results from these prediction models for sex and age. The variance explained (R^2^) was 77% for the sex-based model and 36% for age. We also compared our Lasso models with corresponding sex- and age based models trained on 1,487 randomly selected genome-wide CpGs. These predictions were performed 1,000 times for both sex and age models and R^2^ distributions from the subsequent regression models can be seen in Fig 2C and 2D, respectively. Fig 2C indicates that the prediction model for sex did not perform significantly different, with respect to R^2^, from genome-wide random draws of CpGs (p = 0.09). For age (Fig 2D), we found that R^2^ was actually slightly lower than expected compared to R^2^ from randomly drawn CpGs (p = 0.04). Hence, while we found several sex and age related DMPs linked to genes associated with severe COVID-19 and SARS-CoV-2 host cell entry the proportion did not exceed what would be expected from random genome-wide sampling of CpGs. While we found no differences for sex related DMPs linked to severe COVID-19 and host cell entry connected genes, for age related DMPs the effect was found to be lower than expected. Our findings therefore may suggest that age and sex may not be impossible barriers for the SARS-CoV-2 virus to increase infectiousness [42].

Strengths of our study include the use of a large randomly selected and homogeneous population as well as the use of the Illumina EPIC array with 850K probes. A weakness of this study is the restriction to peripheral blood. A recent study did however find strong associations between DNAm and COVID-19 infection in peripheral blood [43]. Nevertheless, scrutiny of CpGs in cells from the upper and lower respiratory tract is likely to be more revealing in terms of SARS-CoV-2 host cell entry. Another more general weakness is that the effects reported here are likely underestimations as the EPIC array only covers approximately 3% of methylation sites in the human genome [44].

## Conclusions

In summary, we found no association between age and DMPs related to the *ACE2* receptor gene but several DMPs associated with sex. Whether these differences are due to X inactivation bias is not known. Furthermore, we found no hypo-methylated DMPs linked to the *ACE2* TSS, raising the question whether this receptor is at all expressed in peripheral blood. In general, we detected widespread associations between sex and age in CpGs linked to genes associated with host cell entry as well as severe COVID-19. However, these DMPs did not appear to be more strongly associated with sex and age than CpGs drawn genome-wide at random suggesting that age and sex may not be impossible obstacles for the SARS-CoV-2 virus to surmount to increase infectivity.

## Supporting information

S1 Fig A word document containing a PyMOL depiction of the trimer SARS-CoV-2 Spike protein with the subdomains S1, RBD and S2 highlighted in blue, red and orange colors, respectively. (DOCX)

S2 Fig A PDF file with a figure that shows the DNAm density of CpGs linked to the transcription start site (TSS) of the *ACE2* gene in whole blood for males (blue) and females (red). (PDF)

S1 Appendix An Excel file containing all results from the regression analyses together with information regarding all genes included in the study from the STRINGDB web site [26]. (XLSX)
