# Supplementary material for: Age and sex effects on DNA methylation sites linked to genes implicated in severe COVID-19 and SARS-CoV-2 host cell entry
Source: PLoS One. 2022 Jun 9;17(6):e0269105. doi: 10.1371/journal.pone.0269105 (PMC9182232; doi:10.1371/journal.pone.0269105)
Supplement: S1 Fig — (DOCX) [file pone.0269105.s001.docx]

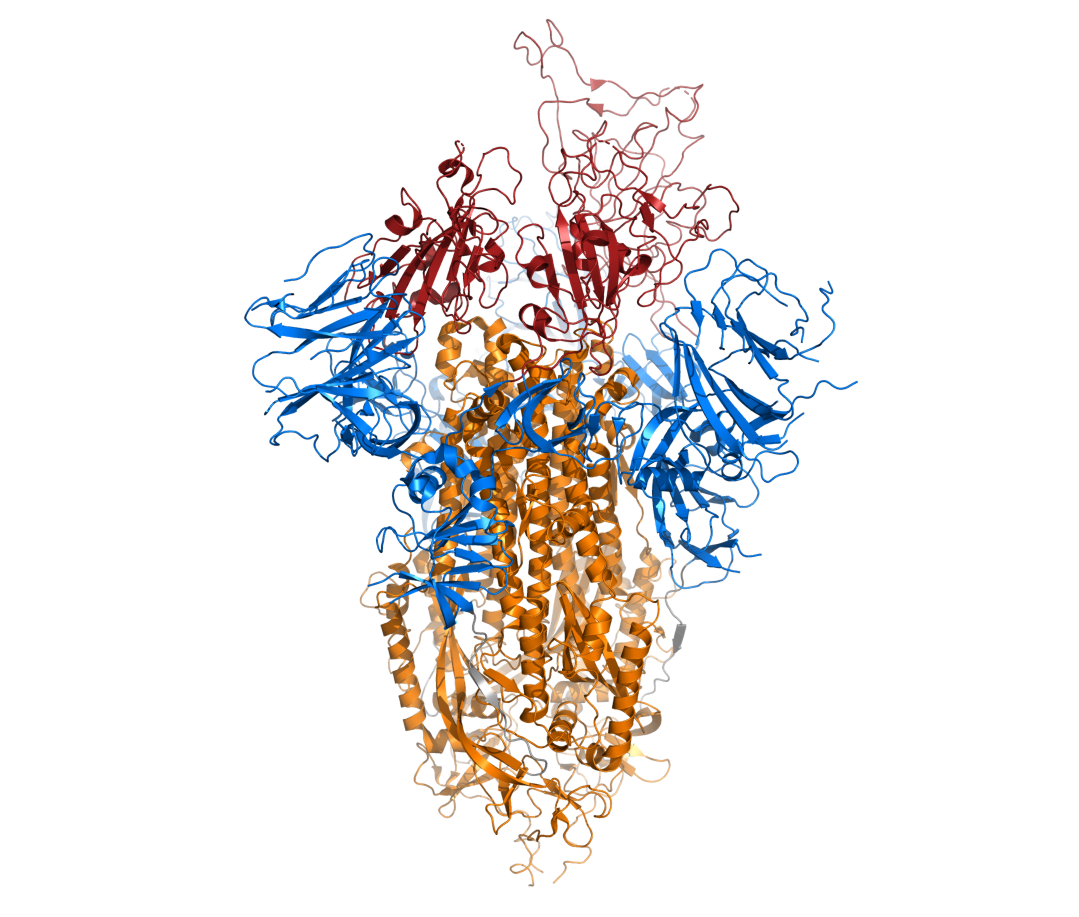


**Figure S1 SARS-CoV-2 Spike protein.**

The figure shows a protein model of the trimeric SARS-CoV-2 Spike protein (NCBI PDB code:6XM0) visualized with the PyMOL Molecular Graphics System Version 2.0 Schrödinger, LLC. The blue regions designate Spike subdomain S1, the red colored regions make up the RBD domain (part of S1) that binds to the *ACE2* receptor. The region colored orange is the S2 subdomain.
