# Supplementary figures and images for: Age and sex effects on DNA methylation sites linked to genes implicated in severe COVID-19 and SARS-CoV-2 host cell entry
Source: PLoS One. 2022 Jun 9;17(6):e0269105. doi: 10.1371/journal.pone.0269105 (PMC9182232; doi:10.1371/journal.pone.0269105)

## ACE2 receptor gene (TSS)

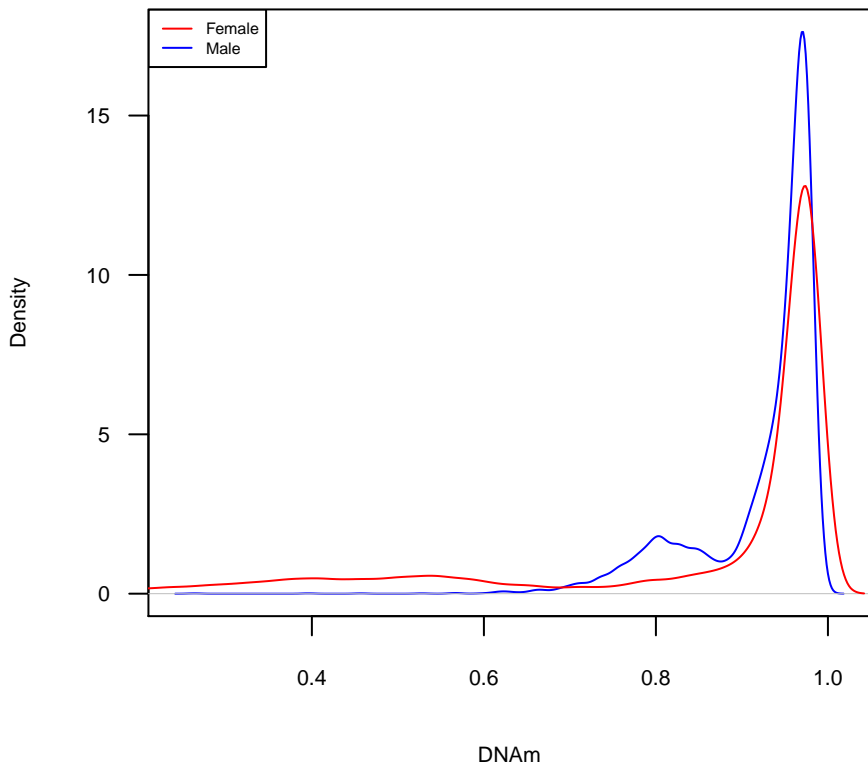

Supplement: S2 Fig — (PDF) [file pone.0269105.s002.pdf]
